# Lithium Induces Oxidative Stress, Apoptotic Cell Death, and G2/M Phase Cell Cycle Arrest in A549 Lung Cancer Cells

**DOI:** 10.3390/molecules30081797

**Published:** 2025-04-17

**Authors:** Pearl Ramushu, Dikgale D. Mangoakoane, Raymond T. Makola, Thabe M. Matsebatlela

**Affiliations:** Department of Biochemistry, Microbiology and Biotechnology, Faculty of Science and Agriculture, University of Limpopo, P/bag x1106, Sovenga 0727, South Africa

**Keywords:** lithium chloride, lung cancer, oxidative stress, apoptosis, cell cycle arrest

## Abstract

Lithium has been identified more than six decades ago as a preferred treatment option for manic depression. Due to its affordability, stability, minimal side effects, and immunomodulatory effects, recent studies on lithium have focused on its potential anticancer properties and possible mechanisms of action. Lung cancer ranks the highest as the main cause of death in males and has high mortality rates with low survival rates. In this study, lung adenocarcinoma (A549) cells were treated with various concentrations of lithium chloride to evaluate its inflammatory and anticancer properties. The in vitro cytotoxic effects of lithium chloride were assessed using the MTT [3-(4, 5-dimethythiazol-2-yl)-2, 5-diphenyltetrazolium bromide] assay, Muse^®^ cell death, and cell cycle analysis. The nitric oxide and oxidative stress flow cytometry Muse^®^ assays were used to monitor inflammation profiles of lithium-treated lung adenocarcinoma cells. The MTT viability assay showed the safe use of LiCl on the noncancerous RAW 264.7 macrophage cells below a concentration of 40 mM. Lithium reduced cell viability, induced late apoptotic cell death, and disrupted normal cell cycle progression in a dose-dependent manner, leading to cell cycle arrest in the S and G2/M phases of A549 cells. The induction of cell death by lithium in A549 cells is accompanied by increased ROS and nitric oxide production. This study shows that lithium chloride possesses some immunomodulatory cytotoxic effects on A549 lung cancer cells and can be further investigated for use in lung cancer treatment.

## 1. Introduction

Lung cancer ranks first as the main cause of cancer morbidity in males and has a strong association with a previous history of smoking [1,2]. Approximately 85% of patients have a group of histological subtypes collectively known as non-small-cell lung cancer (NSCLC), which accounts for 50–60% of all cases [3,4]. Lung cancer has an overall five-year survival rate of 16.8% due to the limited therapeutic options and high levels of tumour metastasis [5]. Hence, it is important to explore new treatment strategies for lung cancer.

Lithium is an FDA-approved and preferred therapy for the treatment of bipolar disorders, manic depression, and numerous psychiatric episodes. Patients on lithium experience modulated immune profiles that include an increase in white blood cell count, which is an observation that could explain the immunoprotective effects that warranted further investigation on its use beyond the treatment of bipolar disorders [6,7,8]. Lithium has been demonstrated to be selectively cytotoxic against several cancer cell lines such as leukaemia [9] and ovarian [10] and colorectal [11] cancers while showing neuroprotective properties in normal neuronal tissue [12,13]. When compared with traditional anticancer therapies, metal ion treatment can kill tumour cells with the elicitation of fewer side effects and less drug resistance [14]. Lithium also regulates major biological processes such as receptor-mediated signalling, ion transport, inflammatory signalling pathways, and reactive oxygen species (ROS) production in various immune cell types [15,16].

The ROS family is a cluster of highly unstable bioactive molecules that are generated during inflammation and normal cellular metabolism via the incomplete reduction of molecular oxygen [17,18]. Their overproduction deregulates the antioxidative defence system and is closely associated with various diseases such as cancer [19]. Likewise, reactive nitrogen species (RNS), the byproducts of nitric oxide (NO) metabolism, play a crucial role in maintaining various physiological functions and contribute to several pathological processes at multiple levels [20]. Various chemotherapeutic drugs cause cells to release NO radicals, which then induce the cytotoxic death of breast, liver, and skin tumours [21].

The induction of cell death is a critical mechanism for a potent anticancer compound. Apoptosis is a type of cell death that can be triggered by mild cellular injury, as well as intracellular and extracellular signals. As a result of apoptosis induction, the damaged cells are then disposed of in an orderly manner without rupturing the cells and exposing the cellular contents to the extracellular environment [22,23]. Apoptosis is regulated by tumour suppressor proteins that include p53 and the Bcl-2 family of proteins which can be either pro-apoptotic or anti-apoptotic [24]. Anticancer therapies, such as chemotherapy, mainly induce cell death by causing either G0/G1 or G2/M cell cycle arrest. Regulation over cell cycle entry and exit is critical because cancerous cells do not appropriately respond to cues that trigger the quiescence phase [25,26]. Hence, there is a need for treatment alternatives that can disrupt and halt the cell cycle of cancerous cells.

In this study, the safety of lithium chloride was tested against the RAW 264.7 cells. The cytotoxicity, reactive oxygen species production, nitric acid production, mode of cell death, and cell cycle arrest potential of lithium chloride were evaluated in lung adenocarcinoma (A549) cells.

## 2. Results

### 2.1. Cytotoxicity of LiCl in RAW 264.7 Cells

**Figure 1:**
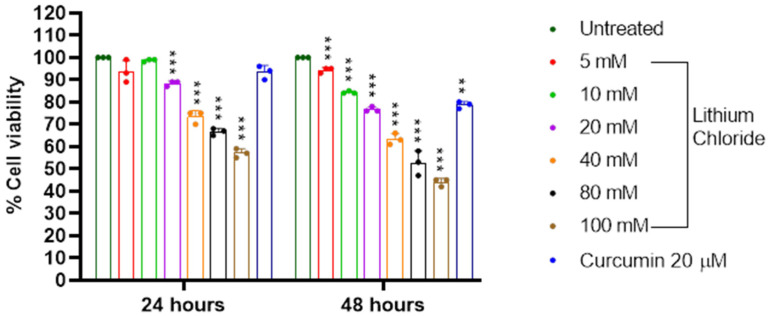
Safety of lithium chloride on RAW 264.7 cells. Cell viability (%) after treatment with varying concentrations of LiCl (0, 5 mM, 10 mM, 20 mM, 40 mM, 80 mM, 100 mM) and curcumin (20 µM) RAW 264.7 macrophage cells over 24 and 48 h. Cell viability was assessed using an MTT assay, and the results show that increasing concentrations of LiCl decreased the cell viability of RAW 264.7 cells. At 24 h, cell viability significantly dropped at 40 mM and higher concentrations, with the most pronounced reduction at 100 mM. At 48 h, the dose-dependent reduction in cell viability became even more apparent, with significant decreases observed at 20 mM and higher concentrations. Statistical significance is indicated as ** *p* < 0.01, and *** *p* < 0.001 compared to the control group. Error bars represent mean + standard deviation.

Figure 1 shows that increasing concentrations of lithium chloride result in a gradual decrease in the cell viability of RAW 264.7 cells over 24 and 48 h. At 24 h, the highest concentration of 100 mM reduced cell viability to 57.0%, while the lowest concentration of 5 mM had a minimal effect (93.667%). A similar trend was observed at 48 h, with 100 mM reducing viability further to 44.0% compared to 94.333% at 5 mM (see Appendix A, Table A1) Curcumin (20 µM) served as a positive control and it maintained higher cell viability compared to most concentrations, showing 93.333% and 78.667% at 24 and 48 h, respectively. The results demonstrate a dose- and time-dependent cytotoxic effect of LiCl in RAW 264 macrophage cells, with greater reductions in cell viability observed at higher concentrations and longer exposure times.

### 2.2. Effect of LiCl on the Cell Viability of A549 Lung Adenocarcinoma Cells

**Figure 2:**
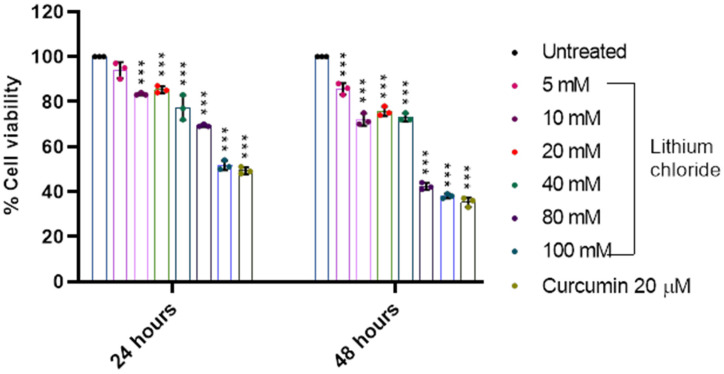
Lithium chloride decreases the cell viability of A549 lung cancer cells. The effect of varying concentrations of lithium chloride (0, 5 mM, 10 mM, 20 mM, 40 mM, 80 mM, 100 mM) and curcumin (20 µM) on cell viability (%) was evaluated after 24 and 48 h of treatment. Cell viability was measured using an MTT assay and data are represented as mean ± standard error of the mean (SEM) from three repeats. Treatment with increasing concentrations of LiCl demonstrated a dose-dependent decrease in cell viability. At 24 h, significant reductions in viability were observed at concentrations ≥ 40 mM, with the lowest viability recorded at 100 mM. After 48 h, the cytotoxic effect of LiCl was more noticeable, with significant decreases beginning at 20 mM. Curcumin (20 µM) exhibited a protective effect (used as a positive control). Statistical significance is indicated as *** *p* < 0.001. Error bars represent mean + standard deviation.

Figure 2 illustrates a dose- and time-dependent reduction in cell viability following treatment with LiCl in A549 cells. At 24 h, cell viability declined gradually with increasing concentrations from 94.0% at 5 mM to 51.667% at 100 mM (see Appendix A, Table A2). A similar trend was observed at 48 h but with more pronounced effects, as viability dropped to 85.667% at 5 mM and as low as 38.0% at 100 mM. Notably, the positive control, curcumin (20 µM), exhibited significant cytotoxic effects, with a cell viability of 49.333% at 24 h and 35.333% at 48 h. Overall, the results demonstrate that LiCl reduces cell viability in a concentration- and time-dependent manner, with higher doses and longer exposure times leading to greater cytotoxicity in A549 cells.

### 2.3. Effect of Lithium on the Production of Reactive Oxygen Species in A549 Cells

**Figure 3:**
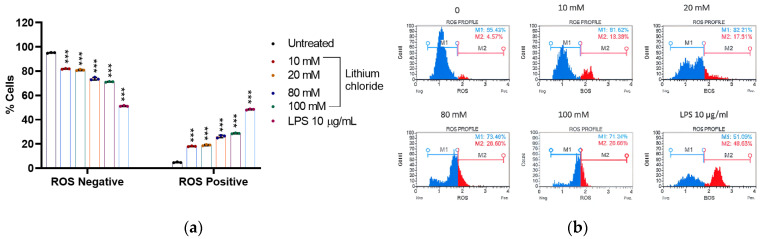
Lithium chloride increases ROS production in A549 cells. (**a**) Percentage of ROS-negative and ROS-positive A549 cells following treatment with lithium chloride at various concentrations (0, 10 mM, 20 mM, 80 mM, 100 mM) and LPS (10 µg/mL). (**b**) ROS profiles of lithium chloride and LPS. These results suggest that LiCl induces oxidative stress in a dose-dependent manner. ROS levels were measured using an oxidative stress flow cytometry Muse^®^ assay, and data are expressed as the mean ± standard error of three repeats. Statistical significance is indicated as *** *p* < 0.001 compared to the untreated control.

Figure 3 reveals a concentration-dependent increase in ROS-positive cells with increasing concentrations of LiCl. In the untreated control, the majority of cells were ROS-negative (95.170%), with only 4.830% being ROS-positive (see Appendix A, Table A3). Treatment with 10 mM and 20 mM LiCl increased the ROS-positive population to 18.067% and 19.043%, respectively. Higher concentrations of 80 mM and 100 mM further elevated ROS-positive cells to 26.200% and 28.727%, respectively, indicating a clear trend of elevated oxidative stress in A549 cells. LPS (10 µg/mL), used as a positive control, caused the most significant increase in ROS-positive cells (48.470%), demonstrating its strong pro-oxidative effect. These results suggest that LiCl induces oxidative stress in a dose-dependent manner, as evidenced by the increase in ROS-positive cells, although its effect is less pronounced than that of LPS. This shows that LiCl contributes to cellular ROS generation at higher concentrations.

### 2.4. Effect of Lithium on the Production of Nitric Oxide in A549 Cells

**Figure 4:**
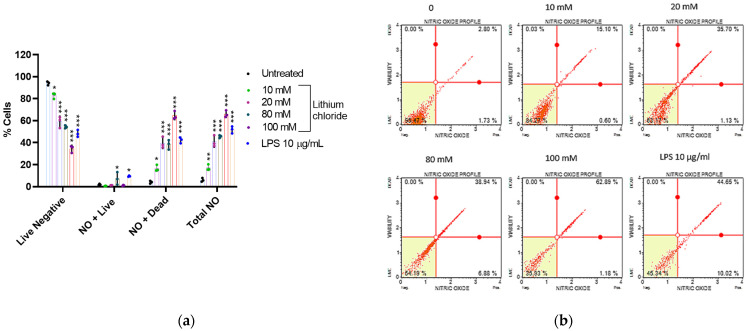
Lithium chloride increases nitric oxide production and lowers cell viability of A549 cells. (**a**) The bar graph represents the percentage of live NO-negative cells, live NO-positive cells, dead NO-positive cells, and total NO-positive cells after treatment with various concentrations of LiCl. (**b**) Nitric oxide profiles of various concentrations of LiCl and LPS. The graph demonstrates that lithium chloride induces nitric oxide (NO) production and affects the cell viability of A549 cells in a dose-dependent manner. Data were determined using DAF2-DA/PI staining for NO detection and flow cytometry. Data are presented as the mean ± standard error of three repeats. Statistical significance is indicated as * *p* < 0.05, ** *p* < 0.01, and *** *p* < 0.001 compared to the untreated control.

Figure 4 shows that LiCl induces a dose-dependent increase in nitric oxide (NO) production and NO-associated cell death in A549 cells. In the untreated control, the majority of cells were live and NO-negative (93.977%), with only 6.023% total NO-positive cells. Treatment with 10 mM and 20 mM LiCl resulted in a substantial reduction in live NO-negative cells (82.840% and 58.980%, respectively) and an increase in dead NO-positive cells (16.540% and 39.680%). At higher concentrations (80 mM and 100 mM), the percentage of live NO-negative cells continued to decline (54.353% and 34.367%, respectively), while dead NO-positive cells rose dramatically, peaking at 64.563% at 100 mM (see Appendix A, Table A4). The total NO-positive population reached 65.970% at 100 mM, indicating robust NO production and associated cytotoxicity. LPS (10 µg/mL), used as a positive control, also increased NO-positive cells (51.753% total NO-positive) with a higher proportion of dead NO-positive cells (42.257%), supporting its role as a potent inducer of NO production and cell death. These findings suggest that LiCl stimulates NO production in a dose-dependent manner, leading to increased cell death, primarily through NO-mediated cytotoxicity. The effects of LiCl are comparable to LPS at higher concentrations, indicating its significant role in inducing oxidative stress and cell death, as seen in Figure 2 and Figure 3.

### 2.5. Mode of Cell Death Induced by LiCl in A549 Cells

**Figure 5:**
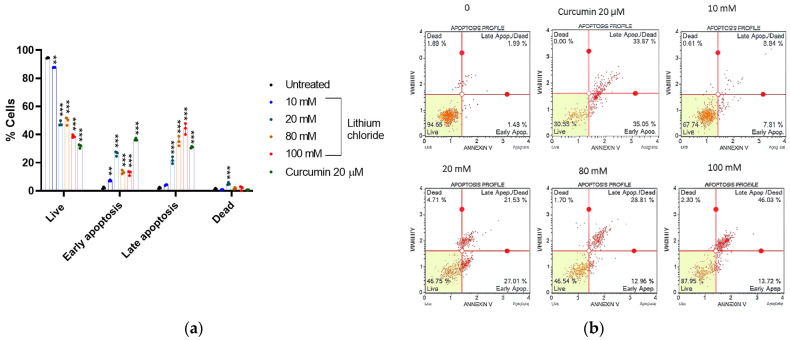
Lithium chloride induces late apoptotic death in A549 cells. (**a**) Distribution of live, early apoptotic, late apoptotic, and dead cells following treatment with lithium chloride. (**b**) Apoptotic profiles of various concertation of LiCl and curcumin. A549 cells were treated with various concentrations of LiCl (10 mM, 20 mM, 80 mM, 100 mM), and curcumin (20 µM) was used as a positive control. The data show that LiCl induces dose-dependent effects on cell viability and apoptosis. The results were obtained using the Muse^®^ Annexin V and Dead Cell Kit, and data are expressed as the mean ± standard error of 3 repeats. Statistical significance is indicated as ** *p* < 0.01, and *** *p* < 0.001 compared to the untreated control.

Figure 5 shows that the majority of the A549 cells were live (94.490%), with minimal early apoptotic (2.067%), late apoptotic (2.073%), and dead cells (1.370%) in the untreated control. As the concentration of LiCl increased, the percentage of live cells decreased dramatically, reaching 38.733% at 100 mM (see Appendix A, Table A5). At 20 mM, there was a marked increase in early apoptotic cells (26.433%) and late apoptotic cells (21.677%), whereas at the higher concentrations (80 mM and 100 mM), the majority were late apoptotic cells, peaking at 44.130% at 100 mM, while early apoptosis decreased slightly. Curcumin (20 µM) caused a significant increase in early apoptosis (36.527%) and a relatively high percentage of late apoptotic cells (31.027%), while dead cells remained low (0.727%). These results suggest that LiCl induces cell death predominantly through apoptosis, with higher concentrations shifting cells toward late apoptotic stages.

### 2.6. Cell Cycle Arrest Potential of Lithium Chloride

**Figure 6:**
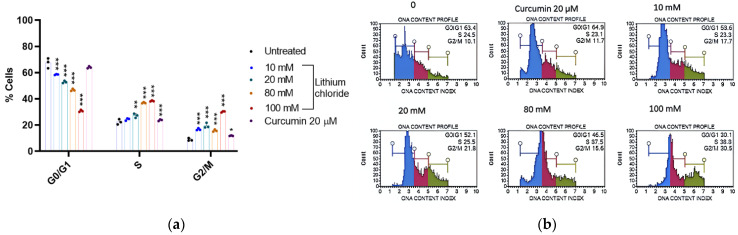
Lithium chloride induces the G2/M phase of A549 cells. (**a**) Distribution of A549 cells in G0/G1, S, and G2/M phases. (**b**) Cell cycle profiles of various LiCl concentrations and curcumin. Cells were treated with LiCl at concentrations of 0, 10 mM, 20 mM, 80 mM, and 100 mM or curcumin, and they were analysed using the Muse^®^ Cell Analyzer. As the concentration of LiCl increased, the percentage of A549 cells in G0/G1 decreased significantly while simultaneously accumulating in the S and G2/M phases. Statistical significance is indicated as * *p* < 0.05, ** *p* < 0.01, and *** *p* < 0.001 compared to the control group. Error bars represent the mean ± standard error of 3 repeats.

Figure 6 shows that lithium chloride induces a concentration-dependent shift in the cell cycle distribution of A549 cells. In the untreated control, the majority of cells were in the G0/G1 phase (67.467%), with fewer in the S (22.400%) and G2/M (8.900%) phases (see Appendix A, Table A6). As the concentration of LiCl increased, the percentage of cells in G0/G1 decreased significantly, reaching 30.767% at 100 mM. Simultaneously, cells accumulated in the S and G2/M phases, with the S phase peaking at 38.367% and G2/M at 30.167% at 100 mM. Curcumin (20 µM) had a milder effect on the cell cycle, maintaining a higher percentage of cells in the G0/G1 phase (64.033%) compared to high concentrations of LiCl. These results suggest that LiCl disrupts the normal cell cycle progression of A549 cells in a dose-dependent manner, leading to cell cycle arrest in the S and G2/M phases. This disruption is indicative of a mechanism of cytotoxicity or growth inhibition caused by lithium chloride.

## 3. Discussion

Macrophages play crucial roles as a primary line of defence against infectious pathogens or foreign agents also by cleaning up apoptotic debris [27]. As such, RAW 264.7 cells were used as a model of macrophages. Figure 1 shows that concentrations of 20 mM and below are significantly less toxic to RAW 264.7 cells after 24 h of treatment. Makola et al., 2020, showed that 50–100 mM lithium chloride concentrations were cytotoxic to RAW 264.7 cells [15]. Several studies have shown that LiCl activates pathways that lead to a macrophage polarisation shift from the pro-inflammatory M1 phenotype to the anti-inflammatory M2 phenotype [28,29]. This reprogramming of macrophages might be useful in reducing the chronic inflammation seen in many cancers.

Lithium is not regulated by any transport or permeation pathways that are selective for other ions, and its therapeutic effects are partly due to its competition with sodium and magnesium, which affects fundamental cellular processes [30]. Our results showed that the cell viability of A549 significantly reduced to 69 and 51% for 80 and 100 mM, respectively, after 24 h of treatment (Figure 2). Lan et al., 2013, showed that the proliferation of A549 cells was significantly inhibited by LiCl at concentrations of above 40 mM [31]. Figure 1 and Figure 2 showed that at 10 mM LiCl, the cell viability remains high in both RAW 264.7 cells and A549 cells. While 20 mM LiCl caused a moderate reduction in the cell viability of both cell lines, it demonstrated slight toxicity to RAW 264.7 cells after 48 hours of treatment. The concentration of ingested lithium is comparable in both intracellular and extracellular compartments [30]; hence, it is important to highlight safety for normal cells. These results show that even low concentrations of LiCl have a noticeable biological effect.

Redox metabolism leads to an abnormal accumulation of reactive oxygen species in cancer cells; therefore, its involvement in the molecular pathways of A549 cells was investigated. ROS can either help prevent cancer by killing abnormal cells or they can promote cancer by causing mutations [32,33]. Treatment with LiCl without any stimuli induced ROS production of 19, 26, and 28% for 20, 80, and 100 mM, respectively (Figure 3). This suggests that LiCl-induced ROS contributes to cell death, possibly by forming nuclear DNA damage. Other studies showed that LiCl exerted pro-oxidant effects in colon cancer cells and human choroidal melanoma cells [11,34].

As another free radical that affects cancer-related processes, nitric oxide was also investigated. Nitric oxide synthase produces relatively large amounts of NO in response to inflammatory or mitogenic stimuli and acts in a host-defensive role through its oxidative toxicity [35,36]. The NO production assay (Figure 4) showed that as the concentration of lithium chloride increases, the percentage of live NO-negative cells significantly decreases, with a corresponding increase in NO-positive populations. At 10 mM, the percentage of dead NO-positive cells was relatively low, but at 20 mM and higher concentrations (80 mM and 100 mM), there was a substantial increase in dead NO-positive cells, particularly at 100 mM. These findings align with the known cytotoxic effects of excessive nitric oxide inducing cell damage and apoptosis [37].

Figure 5 shows that high concentrations of lithium chloride induce late apoptosis in A549 cells. Late apoptotic cells that have lost membrane integrity release inflammatory intracellular contents. However, at a concentration of 20 mM, there was a marked increase in early apoptotic cells. Figure 3 and Figure 4 showed that lithium chloride promotes oxidative stress and NO production in A549 cells, which then leads to cell death as observed. Other studies have shown that LiCl induces apoptosis with DNA fragmentation in various types of cancer cells, including schwannoma cells [38], glioblastoma [39], breast cancer [40], and leukaemia [41].

One of the ways to screen for potentially therapeutic drugs is to measure changes in cell cycle kinetics under varying conditions. The cell cycle describes a process that leads to replication and cell division, including G0/G1, the G1/S (synthesis) DNA damage checkpoint, and the G2/M (mitosis) surveillance checkpoints [42]. Lithium’s competition with sodium and magnesium in tumour cells disrupts sodium gradients, and magnesium-dependent enzymatic processes can lead to cell cycle arrest [43]. An article by Chen et al. (2004) demonstrated lithium-induced G2/M arrest in pig airway epithelial cells at 10 mM [44]. The pig airway epithelial cells offer a physiologically relevant model for normal lung tissue; hence, the influence of LiCl on lung adenocarcinoma cells is worth investigating. Based on the results shown in Figure 6, concentrations of 10 and 20 mM start disrupting the cycle, and higher concentrations (80–100 mM) display strong arrest in the S and G2/M phases, which can prevent proliferation and lead to cell death. The ability of LiCl to affect the cell cycle of both non-cancerous (pig airway epithelial cells) and cancerous (lung adenocarcinoma cells) cells highlights its action on shared cellular processes. While this activity can support the use of lithium chloride as a cytostatic agent, it also highlights the importance of balancing efficacy with potential toxicity, especially around fast-dividing normal tissue. Other studies showed that lithium arrests cells in the S and G2/M phases in both leukaemia [11] and glioblastoma [45] cells but only the G2/M phase in oesophageal cells [46].

A study by Ma et al. (2024) uncovered a promising function of lithium carbonate in the metabolic reprogramming of T cells, thus allowing T cells to overcome immunosuppression and restore their cytotoxicity, a new angle for enhancing anti-tumour immunity [47]. Human lung tumour cells can produce 40 times more lactic acid than normal cells, and this utilisation of lactic acid as fuel adds to the growing evidence of lithium’s potential anticancer properties [47,48]. The results of this study showed that lithium chloride increases oxidative stress, which may make lactate-rich cells more vulnerable, thus leading to apoptosis. Lung adenocarcinoma cells undergoing cell cycle arrest and cell death at lower LiCl concentrations provide a potential therapeutic window and potential effects on the tumour immune system [49].

## 4. Materials and Methods

### 4.1. Cell Line and Culture

The lung adenocarcinoma A549 cells (ATCC^®^ CCL-185TM) and RAW 264.7 cells (ATCC^®^ TIB-71™) were maintained in cell culture flasks at 37 °C in a humidified 95% air and 5% CO2 incubator. The A549 cells were maintained in Roswell Park Memorial Institute-1640 (RPMI-1640) medium (HyClone™, Logan, UT, USA) supplemented with 1% penicillin/streptomycin/neomycin (PSN) [Gibco, Auckland, New Zealand] and 10% heat-inactivated foetal bovine serum (FBS, Gibco, Auckland, New Zealand). The RAW 264.7 cells were maintained in Dulbecco’s Modified Eagle Medium (DMEM) [HyClone™, Logan, UT, USA] supplemented with 1% PSN (Gibco, Auckland, New Zealand) and 10% heat-inactivated FBS (Gibco, Auckland, New Zealand).

After reaching 80–90% confluency, the cells were washed to remove FBS and were trypsinized using 1 mL of 1% trypsin. Trypsin was deactivated by adding a culture medium containing FBS. The cells were plated to set up for a specific experimental treatment plan. For an experimental setup, the cells were seeded in a 6-well plate at a concentration of 2 × 10^5^ cells/mL and incubated overnight in a CO_2_ incubator. Thereafter, the cells were treated with various concentrations (10, 20, 40, 80, and 100 mM) of lithium chloride (LiCl). Non-treated control cells and a positive control treated with 20 μM curcumin (Sigma, St. Louis, MO, USA) and 10 μg/mL LPS (Sigma, St. Louis, MO, USA) were included in respective experiments.

### 4.2. Cell Viability Assay

The cytotoxicity of lithium chloride was assessed using the MTT [3-(4, 5-dimethylthiazol-2-yl)-2, 5-diphenyltetrazolium bromide] assay, which evaluates cell viability by measuring the oxidative reduction potential of cells, whose outcomes can be quantified spectrophotometrically as the metabolically active cells reduce the yellow MTT into purple formazan and the intensity of the purple colour is directly proportional to the number of viable cells. After 24 and 48 h incubations, the medium was aspirated and 5 mg/mL of MTT (Fluka BioChemika, St. Gallen, Switzerland) was used for 4 h incubation. After incubation, 100 μL of DMSO (Saarchem, Randburg, RSA) was added to each well, and the plates were incubated for an hour. The absorbance was measured at 570 nm using a GloMax Multi microplate reader (Promega, Madison, WI, USA).

### 4.3. Oxidative Stress Assay

The Muse Oxidative Stress reagent is based on dihydroethidium and was used to distinguish live ROS-negative cells from cells exhibiting ROS. To quench out any residual activity, cells were treated with 0.01% sodium borohydride (in PBS) for 5 min before treatment with lithium chloride. The cells were then harvested and stained with the oxidative stress reagent according to the manufacturer’s instructions. The results were then analysed using the Muse^®^ Cell Analyzer (Merck Millipore, Darmstadt, Germany).

### 4.4. DAF2-DA Nitric Oxide Measurement Assay

DAF2-DA (4,5 diaminofluorescein diacetate) is a fluorescein derivative that reacts with an oxidation product of NO to the highly fluorescent triazolofluorescein DAF-2T (Sigma-Aldrich, St. Louis, MO, USA). To quench out any residual activity, cells were treated with 0.01% sodium borohydride (in PBS) for 5 min before treatment with lithium chloride. The cells were harvested after a 24 h treatment. The cells were stained with 10 μM/mL DAF2-DA and 10 μg/mL PI (propidium iodide, Sigma-Aldrich, St. Louis, MO, USA) for 30 min at room temperature. The results were viewed using the Muse^®^ Cell Analyzer (Merck Millipore, Darmstadt, Germany).

### 4.5. Annexin-V and (PI) Apoptosis Detection Assay

The mode of death induced by lithium chloride was determined using the Muse^®^ Annexin V and Dead Cell Kit. As per the manufacturer’s manual, the kit is used to differentiate live, apoptotic, and dead cells with Muse^®^ Annexin V and Dead Cell Reagent. The samples were harvested and stained for 20 min at room temperature in the dark. The results were viewed using the Muse^®^ Cell Analyzer (Merck Millipore, Darmstadt, Germany).

### 4.6. Cell Cycle Arrest Assay

The ability of lithium chloride to induce cell cycle arrest was determined using the Muse^®^ Cell Cycle Kit, which allows for fast quantitative measurements of the percentage of cells in the G0/G1, S, and G2/M phases of the cell cycle. The A549 cells were treated and harvested as mentioned above. Then, they were stained with the Muse^®^ Cell Cycle Reagent, followed by incubation for 30 min at room temperature in the dark. The results were viewed using the Muse^®^ Cell Analyzer (Merck Millipore, Darmstadt, Germany).

### 4.7. Statistical Analysis

All the experiments were performed in triplicate in three independent experiments, and the results were presented as mean ± standard deviation (SD). The experimental data were analysed using GraphPad Prism Version 6.0 Statistical Software. Statistical significance was determined by a one-way analysis of variance (ANOVA) and the Tukey–Kramer multiple comparisons test. The *p* values less than 0.01 were considered significant (* *p* < 0.05, ** *p* < 0.01, *** *p* < 0.001).

## 5. Conclusions

Results from this study suggest that increased ROS generation and oxidative stress have a crucial role to play in lithium cytotoxic mechanisms in A549 lung cancer cells. While the concentration of 100 mM of lithium chloride showed the best results in all assays, we advise for the use of lower concentrations to avoid any toxicities as observed in the 264.7 macrophage cells at higher concentrations. The concentration of 10 mM is less likely to harm healthy cells while being selectively cytotoxic to the highly proliferating A549 lung cancer cells. Further studies are required to investigate the effect of lithium on cytokine production, cell cycle gene expression patterns, and apoptosis-related gene expression in A549 lung cancer cells in relation to oxidative stress and ROS levels. This study provides a basis for considering lithium as a therapeutic agent for use in cancer treatment in addition to its use in the management of bipolar disorders.

## Data Availability

The original contributions presented in this study are included in the article. Further inquiries can be directed to the corresponding author.

## Abbreviations

The following abbreviations are used in this manuscript:

LiCl	Lithium chloride
FDA	Food and Drug Administration
ROS	Reactive oxygen species
NO	Nitric oxide
DNA	Deoxyribonucleic acid
LPS	Lipopolysaccharide

## Appendix A

**Table A1:** Effect of varying concentrations of lithium chloride and curcumin (20 µM) on cell viability (%) over 24 and 48 h. Values are expressed as mean ± standard deviation (*n* = 3).

	24 h	48 h
0	100.000 ± 0.000	100.000 ± 0.000
5 mM	93.667 ± 5.033	94.333 ± 1.1555
10 mM	98.667 ± 0.577	84.333 ± 0.577
20 mM	88.333 ± 1.155	76.667 ± 1.155
40 mM	73.333 ± 2.887	63.333 ± 2.517
80 mM	66.667 ± 1.528	52.667 ± 5.508
100 mM	57.000 ± 2.000	44.000 ± 1.732
Curcumin 20 µM	93.333 ± 3.055	78.667 ± 1.528

**Table A2:** Effect of varying concentrations of lithium chloride and curcumin (20 µM) on cell viability (%) over 24 and 48 h. Cell viability was assessed using an MTT assay, and the results are expressed as mean ± standard deviation (*n* = 3).

	24 h	48 h
0	100.000 ± 0.000	100.000 ± 0.000
5 mM	94.000 ± 3.606	85.667 ± 2.516
10 mM	83.333 ± 0.577	72.000 ± 2.646
20 mM	85.333 ± 1.528	75.667 ± 2.082
40 mM	77.333 ± 5.508	73.000 ± 1.732
80 mM	69.333 ± 0.577	42.333 ± 1.528
100 mM	51.667 ± 2.082	38.000 ± 1.000
Curcumin 20 µM	49.333 ± 1.528	35.333 ± 2.082

**Table A3:** Effect of LiCl at various concentrations (0, 10 mM, 20 mM, 80 mM, 100 mM) and LPS (10 µg/mL) on the percentage of ROS-negative and ROS-positive cells. ROS levels were determined using a Muse ROS assay, and results are expressed as mean ± standard deviation (*n* = 3).

	ROS Negative	ROS Positive
0	95.170 ± 0.352	4.830 ± 0.352
10 mM	81.933 ± 0.326	18.067 ± 0.326
20 mM	80.957 ± 0.531	19.043 ± 0.531
80 mM	73.800 ± 1.087	26.200 ± 1.087
100 mM	71.243 ± 0.326	28.727 ± 0.365
LPS 10 μg/mL	51.303 ± 0.396	48.470 ± 0.452

**Table A4:** Effect of lithium chloride on nitric oxide (NO) production and cell viability of A549 cells. The table shows the percentage of live NO-negative cells, live NO-positive cells, dead NO-positive cells, and total NO-positive cells (% total nitric oxide) after treatment with LiCl at concentrations of 10 mM, 20 mM, 80 mM, and 100 mM or LPS (10 µg/mL, positive control). Data were determined using DAF2-DA/PI staining for NO detection and flow cytometry and are expressed as mean ± standard deviation (*n* = 3).

	% Live Negative	% Nitric Oxide + Live	% Nitric Oxide + Dead	% Total Nitric Oxide
0	93.977 ± 1.698	1.943 ± 0.580	4.080 ± 1.237	6.023 ± 1.698
10 Mm	82.840 ± 2.705	0.610 ± 0.185	16.540 ± 2.875	17.150 ± 2.713
20 mM	58.980 ± 5.172	1.220 ± 0.085	39.680 ± 5.130	40.900 ± 5.209
80 mM	54.353 ± 1.382	7.343 ± 5.699	38.287 ± 4.317	45.543 ± 1.297
100 mM	34.367 ± 3.305	1.073 ± 0.459	64.563 ± 3.760	65.970 ± 3.119
LPS 10 μg/mL	48.250 ± 3.123	9.497 ± 0.482	42.257 ± 2.668	51.753 ± 3.128

**Table A5:** Effects of lithium chloride on apoptosis in A549 cells. The table shows the percentage of live, early apoptotic, late apoptotic, and dead cells after treatment with LiCl (0, 10 mM, 20 mM, 80 mM, and 100 mM) and curcumin (20 µM). Data were obtained from Annexin V/PI staining and then analysed using the Muse^®^ Cell Analyzer and are expressed as mean ± standard deviation (*n* = 3).

	% Live	% Early Apoptotic	% Late Apoptotic	% Dead
0	94.490 ± 0.204	2.067 ± 0.634	2.073 ± 0.471	1.370 ± 0.506
10 mM	87.723 ± 0.029	7.257 ± 0.479	4.190 ± 0.303	0.833 ± 0.194
20 mM	47.867 ± 1.781	26.433 ± 1.652	21.677 ± 2.423	5.027 ± 0.898
80 mM	49.613 ± 2.747	13.270 ± 1.352	35.280 ± 3.381	1.843 ± 0.569
100 mM	38.733 ± 1.021	12.633 ± 1.613	44.130 ± 3.928	1.840 ± 1.456
Curcumin 20 µM	31.717 ± 1.293	36.527 ± 0.826	31.027 ± 0.544	0.727 ± 0.006

**Table A6:** Cell cycle distribution (%) of A549 cells after treatment with lithium chloride at various concentrations (0, 10 mM, 20 mM, 80 mM, 100 mM) and curcumin (20 µM). Data represent the percentage of cells in the G0/G1, S, and G2/M phases of the cell cycle, measured by the Muse^®^ Cell Analyzer. Values are expressed as mean ± standard deviation (*n* = 3).

	G0/G1	S	G2/M
0	67.467 ± 3.790	22.400 ± 2.007	8.900 ± 1.044
10 mM	58.533 ± 0.306	24.333 ± 0.907	16.767 ± 0.814
20 mM	52.867 ± 0.862	27.200 ± 1.609	19.533 ± 1.973
80 mM	46.800 ± 0.889	36.933 ± 0.493	15.767 ± 0.862
100 Mm	30.767 ± 0.764	38.367 ± 0.379	30.167 ± 0.493
Curcumin 20 µM	64.033 ± 0.777	23.767 ± 0.611	11.833 ± 0.231
